# Efficacy of Pimobendan in the Prevention of Congestive Heart Failure or Sudden Death in Doberman Pinschers with Preclinical Dilated Cardiomyopathy (The PROTECT Study)

**DOI:** 10.1111/j.1939-1676.2012.01026.x

**Published:** 2012-10-18

**Authors:** NJ Summerfield, A Boswood, MR O'Grady, SG Gordon, J Dukes-McEwan, MA Oyama, S Smith, M Patteson, AT French, GJ Culshaw, L Braz-Ruivo, A Estrada, ML O'Sullivan, J Loureiro, R Willis, P Watson

**Affiliations:** North Downs Specialist ReferralsBletchingley, Surrey, RH1 4QP, UK; Department of Veterinary Clinical Sciences, The Royal Veterinary CollegeHertfordshire, UK; Ontario Veterinary College, University of GuelphOntario, Canada; College of Veterinary Medicine and Biomedical Sciences, Texas A&M UniversityCollege Station, Texas; School of Veterinary Science and Department of Musculoskeletal Biology, University of LiverpoolNeston, UK; Department of Clinical Studies-Philadelphia, School of Veterinary Medicine, University of PennsylvaniaPhiladelphia, Pennsylvania; Sarah Smith CardiologyEtwall, Derbyshire, UK; HeartVets @ Vale Referrals, The Animal HospitalStinchcombe, Dursley, Glos, UK; Royal (Dick) School of Veterinary Studies, University of Edinburgh and Roslin InstituteScotland, UK; Small Animal Clinical Sciences, University of Florida College of Veterinary MedicineFlorida; Dogs & Cats Veterinary Referral and ERBowie, Maryland; Holter Monitoring ServiceDollar, Clackmannanshire, Scotland, UK; Boehringer Ingelheim Animal HealthIngelheim am Rhein, Germany

**Keywords:** Cardiology, Cardiovascular, Clinical trials, Evidence based medicine, Survival, Therapy

## Abstract

**Background:**

The benefit of pimobendan in delaying the progression of preclinical dilated cardiomyopathy (DCM) in Dobermans is not reported.

**Hypothesis:**

That chronic oral administration of pimobendan to Dobermans with preclinical DCM will delay the onset of CHF or sudden death and improve survival.

**Animals:**

Seventy-six client-owned Dobermans recruited at 10 centers in the UK and North America.

**Methods:**

The trial was a randomized, blinded, placebo-controlled, parallel group multicenter study. Dogs were allocated in a 1:1 ratio to receive pimobendan (Vetmedin capsules) or visually identical placebo.

The composite primary endpoint was prospectively defined as either onset of CHF or sudden death. Time to death from all causes was a secondary endpoint.

**Results:**

The proportion of dogs reaching the primary endpoint was not significantly different between groups (*P* = .1). The median time to the primary endpoint (onset of CHF or sudden death) was significantly longer in the pimobendan (718 days, IQR 441–1152 days) versus the placebo group (441 days, IQR 151–641 days) (log-rank *P* = 0.0088). The median survival time was significantly longer in the pimobendan (623 days, IQR 491–1531 days) versus the placebo group (466 days, IQR 236–710 days) (log-rank *P* = .034).

**Conclusion and Clinical Importance:**

The administration of pimobendan to Dobermans with preclinical DCM prolongs the time to the onset of clinical signs and extends survival. Treatment of dogs in the preclinical phase of this common cardiovascular disorder with pimobendan can lead to improved outcome.

## Introduction

Dilated cardiomyopathy (DCM) is the most common form of cardiomyopathy in dogs and the 2nd most common form of acquired heart disease in dogs after myxomatous mitral valve disease (MMVD). The prevalence of DCM in Doberman Pinschers (Dobermans) increases with age^1^ and the estimated proportion of purebred Dobermans that develop DCM in their lifetime is approximately 25–50%.^1^,^2^ The clinical stage of DCM is typically characterized by signs of congestive heart failure (CHF), with or without cardiac arrhythmias.

DCM in Dobermans is characterized by a protracted preclinical stage (>2–3 years).^3^ During the preclinical stage, progressive left ventricular systolic dysfunction and dilatation develop with or without clinically important ventricular and supraventricular arrhythmias. These arrhythmias often progress in severity. Sudden death caused by ventricular tachyarrhythmia-fibrillation occurs before the onset of CHF in at least 25–30% of affected Dobermans.^4^,^a^,^b^ with a case fatality rate of at least a 90% after 1 year.^3^

Therapies that prolong the preclinical stage of DCM could therefore be of great potential benefit. In humans with preclinical left ventricular systolic dysfunction, treatment with angiotensin converting enzyme (ACE) inhibitors and beta adrenergic receptor blockers is routinely recommended.^6^ The efficacy of prophylactic treatment in Dobermans remains unclear with 1 retrospective study reporting that benazepril might delay the progression of preclinical DCM in Dobermans.^7^ However, despite the result of this retrospective study and the recommendations for treatment of human patients, ACE inhibitors are not widely prescribed in the preclinical stage of DCM, and so affected Dobermans typically remain untreated until CHF or other clinical signs develop.

Previous studies have suggested that pimobendan treatment significantly reduces case fatality and morbidity in Dobermans with CHF secondary to DCM.^8^,^9^ However, the potential benefit of pimobendan treatment in delaying the progression of preclinical DCM in Dobermans has not previously been evaluated. Pimobendan is a benzimidazopyridazinone with a potent positive inotropic^10^ and a vasodilatory^11^ effect. This combined effect of preload and afterload reduction, together with positive inotropic support, could result in a reduction in cardiac size and filling pressures in Dobermans with preclinical DCM. Such beneficial effects could result in prolongation of the preclinical stage of DCM.

The primary objective of this study was to prospectively evaluate whether the chronic oral administration of pimobendan in Dobermans with preclinical DCM could delay the onset of CHF or sudden death. Further aims of the study were to evaluate the effect of pimobendan treatment on time to death (all cause), frequency of ventricular arrhythmia, and heart size.

## Materials and Methods

### Dogs

Client-owned Dobermans were recruited by cardiologists at 10 centers: 5 in the United Kingdom, 4 in the United States, and 1 in Canada. Recruitment occurred initially in the United Kingdom and Canada. A protocol amendment including the addition of 4 centers in the United States was made 24 months after recruitment began. This followed the approval of pimobendan (Vetmedin)^c^ for the treatment of CHF secondary to DCM and MMVD in that country. This amendment was implemented to increase enrollment. In each country, owners were invited to present apparently healthy Dobermans for screening to determine their eligibility for study participation.

### Enrollment Criteria

#### Inclusion Criteria

Dogs were eligible for participation in the study provided that the owner had given informed consent. Dobermans of either sex were included if they were aged between 4 and 9 years (48–119 months inclusive) and had echocardiographic evidence of preclinical DCM, defined according to weight-adjusted values of left ventricular internal dimension in systole (LVIDS) equal to or exceeding those in Table 1. Dogs screened in Texas were only eligible for inclusion if they were negative on an immunofluorescent antibody test against *Trypanosoma cruzi*^d^ at the start of the study.

**Table 1:** Weight-dependent values for left ventricular internal diameter in systole above which dogs were considered to have met the inclusion criterion. LVIDS, left ventricular internal diameter in systole. (Adapted for use in this study from unpublished observations on 51 normal Doberman Pinschers. The study conducted by one of the authors [MOG] assessed the relationship between left ventricular dimension and body weight resulting in the equation; predicted LVIDS equals 0.1402 × BW + 26.7 mm.)

Body Weight (kg) up to:	LVIDS ≥ (mm)
25	38.8
30	39.5
35	40.2
40	40.9
45	41.6
50	42.3

#### Exclusion Criteria

Dogs were ineligible for inclusion in the study if they had any of the following: other cardiac disease (congenital or acquired); current or previous clinical signs attributable to DCM; an episode of syncope within the previous 6 months; sustained (≥30 seconds) ventricular tachycardia based on evaluation of a preinclusion 24-hour ambulatory ECG (Holter); atrial fibrillation; evidence of other clinically important systemic disease; received cardiovascular or respiratory medication(s) including corticosteroids within the previous 6 months; or undergone a general anesthetic in the previous 28 days. In addition, female dogs that were pregnant, lactating, or intended for breeding could not be included.

Because of the potential effect of inadequately controlled hypothyroidism on systolic function,^12^ dogs with known hypothyroidism were initially excluded from the study. A protocol amendment was made after 38 months of the study and subsequently hypothyroid dogs were considered eligible for inclusion provided they met the following criteria: the dog was free from clinical signs of hypothyroidism at the time of inclusion; a thyroxine (T_4_) concentration, measured 4–6 hours after levothyroxine administration, was in the reference range for that particular laboratory; the dose of thyroid supplementation had not been changed in the 60 days before inclusion; and left ventricular systolic dilatation (Table 1) persisted despite adequate thyroxine supplementation.

### Study Design

The trial was a randomized, blinded, placebo-controlled, parallel group, multicenter study. Dogs were allocated in a 1:1 ratio to receive pimobendan (Vetmedin capsules) or visually identical placebo.

The trial protocol was prepared by independent cardiologists in conjunction with the sponsor and was approved by an ethical review committee at all sites where this was required.

The contract between the investigators and the sponsor stipulated that the former group have full access to all results and the right to independently publish results of the study regardless of the trial outcome.

### Randomization and Allocation

Dogs were randomized in pairs over all study sites and over the whole study. Randomization was stratified both by sex (male or female) and the number of ventricular premature complexes (VPCs) on a 3-minute electrocardiogram (ECG) trace (<4 VPCs or ≥4 VPCs). The randomization sequence was generated as a single list, based on Study Case Number, which was held by a single-centralized allocation center. The allocation center was not at a study center. Following the protocol amendment allowing hypothyroid dogs to be recruited to the study, a separate, similarly stratified, randomization list was prepared for these dogs. Once a dog fulfilled the inclusion criteria, that center's dispenser telephoned the central allocation center, and was assigned the next available Study Case Number from the randomization list. The dispenser was also instructed as to which numbered clinical supplies should be used for that case, by a numerical code on the label of each container of trial medication. The role of the dispensers was restricted to the dispensing of appropriate clinical supplies to dog owners, and the dispenser could identify only whether a particular case was in “Treatment Group 1” or “Treatment Group 2.” Investigators, owners, and study monitors had no knowledge of allocations to treatments.

### Blinding

The blinding code for the treatment groups was held by one office and these individuals had no other role in the study. Predefined procedures were available to permit unblinding of individual cases in the event of medical emergency. Unblinding could be achieved by contacting named individuals who held the randomization list; they could then break the treatment code and inform the investigator of the treatment the animal was receiving. Neither the investigators, nor the monitor, nor the sponsor of the study had access to the randomization list.

Pimobendan and placebo were supplied as visually identical capsules and supplied in identical white containers. Containers bore unique numerical codes that enabled dispensers to distinguish between treatment groups, but could not identify which treatment group was receiving pimobendan or placebo. Investigators and owners were unable to distinguish between treatments in any way.

### Test Treatments

Both pimobendan (Vetmedin capsules) and the visually identical placebo were administered at the same dose according to manufacturer's recommendations.^e^ All dogs ≥35.1 kg received 2 × 5 mg pimobendan capsules twice daily (q12h) per os, or the equivalent number of placebo capsules. All dogs ≤35.0 kg received 1 × 5 mg capsule q12h or equivalent placebo. Owners were instructed to administer the test agent in 2 doses per day, approximately 12 hours apart, and at least 1 hour before feeding.^e^ Where animals' weight changed during the study, doses were appropriately adjusted during the study.

### Concomitant Treatments

Administration of nonsteroidal anti-inflammatory drugs and antibiotics was permitted if deemed necessary by the investigator during the blinded portion of the study. Hypothyroid dogs were permitted to receive levothyroxine. If an investigator identified the development of ventricular arrhythmias during the study requiring antiarrhythmic treatment, sotalol treatment was permitted in the protocol. In the event that the arrhythmia was not responsive to sotalol, mexiletine was added. The necessity for treatment was judged by individual investigators. Dogs receiving sotalol with or without mexiletine remained in the study until the primary endpoint was reached. After the primary endpoint was reached, surviving dogs received open label pimobendan (Vetmedin) in addition to any other treatment deemed appropriate by the investigator.

### Schedule of Events

Apparently healthy Dobermans were screened to determine whether they were eligible for study inclusion. All tests were carried out on nonsedated dogs. Before screening was undertaken, a complete history was obtained from the owner to ascertain whether any exclusion criteria were met. Eligible dogs underwent a physical examination (including body weight, kg), 3-minute ECG recording, echocardiogram, Holter, routine hematology, and blood biochemistry including total T_4_, and dogs in Texas also had an immunofluorescent antibody test against *Trypanosoma cruzi* performed.^b^ In cases suspected of hypothyroidism based on a low or normal total T_4_, a free T_4_ by dialysis, thyroid stimulating hormone, or both were measured to confirm the diagnosis. Examinations before inclusion were undertaken between 0 and 14 days (−14 to 0 days) before receiving test medication. The inclusion date was taken to be the day on which the dog first received test medication (day 0). For dogs included in the study, a physical examination, echocardiogram, Holter and routine hematology, and blood biochemistry were repeated in 1–2 months. Follow-up physical examinations were subsequently carried out 6 months after enrollment, every 6 months thereafter, and at study end.

The 3-minute ECG trace was performed, with each dog lying in right lateral recumbency. The total number of VPCs on this ECG trace was recorded. The presence of arrhythmias other than VPCs was noted. Dogs that showed any arrhythmia, other than sinus arrhythmia, during the 3-minute ECG trace were recorded as “arrhythmia yes.” Dog with no evidence of arrhythmia were recorded as “arrhythmia no.” Dogs with a total of 4 or more VPCs during the 3-minute ECG trace were recorded as “VPC ≥ 4,” dogs with fewer VPCs during the 3-minute ECG were recorded as “VPC < 4.”

Echocardiography was performed in a standard manner.^13^ The preinclusion echocardiographic examination (Day −14 to Day 0) consisted of a two-dimensional (2D), M-mode, and Doppler examination to rule out other congenital or acquired cardiac disease (other than DCM). M-mode left ventricular dimensions in systole (LVIDS) and diastole (LVIDD) were acquired from the right parasternal short axis view of the heart at the level of the tips of the papillary muscles. The scheduled follow-up echocardiographic examination included M-mode-derived short axis left ventricular dimensions. All recorded left ventricular dimensions represented the mean of 5 individual measurements (consecutive normal cycles when possible).

A Holter was recorded as part of the preinclusion examination to ensure no dog had sustained ventricular tachycardia (>30 sec). A 2nd Holter was recorded to re-evaluate rhythm, monitor for the development of new arrhythmias, and determine if there was a change in the number of VPCs after initiation of test medication. In the United Kingdom, all Holter recordings were evaluated by one of the authors (RW).^f^ In Canada and the United States, all Holter recordings were analyzed by an experienced technician and the results were validated by another of the authors (MOG). Parameters reported included number of VPCs in 24 hours, presence and frequency of complex ventricular arrhythmias (ie, ventricular pairs, triplets, salvos, or duration of any episodes of sustained ventricular tachycardia: rate >180 beats per minute), paroxysmal atrial fibrillation, or both.

Further diagnostic tests could be undertaken at any time if they were considered indicated by an investigator.

All suspected adverse reactions that occurred during the study were recorded and reported in accordance with local regulations.

### Endpoints

Dogs were considered to have reached the primary endpoint of the study when CHF or sudden cardiac death occurred. A diagnosis of CHF was wherever possible supported by evidence from a thoracic radiograph demonstrating pulmonary edema. All dogs that died unexpectedly with no other apparent cause for death were assumed to have experienced sudden cardiac death. If a dog's clinical condition precluded obtaining thoracic radiographs, a clinical diagnosis of CHF was made based on the opinion of the investigator.

Dogs that died or were euthanized for noncardiac reasons or dogs that were withdrawn from the study for other reasons before reaching the primary endpoint were included in the primary endpoint analysis until they were removed from the study at which point they were censored. Dogs that died for noncardiac reasons were included in the all cause mortality analysis, from which only dogs removed from the study for other reasons or those still alive at the end of the study were censored.

Dogs that had met the primary endpoint of the study and remained alive for a sufficient time to receive oral medication were offered open-label pimobendan (Vetmedin) in addition to standard heart failure treatment and followed until they died or the end of study. There was no standardized treatment or follow-up during this open-label phase of the study. Date of death was recorded as a secondary endpoint. The secondary endpoint was analyzed as all-cause mortality at the end of the study. This endpoint was not predefined in the protocol.

### Outcome Measures

The primary outcome measure of the study was the time to the primary endpoint measured from the day of beginning the study medication (day 0). The secondary outcome measure of all-cause mortality was the time (days) from day 0 to the date of death attributable to any cause.

The change from before enrollment to re-evaluation (1–2 months) in LVIDS and LVIDD and the change in frequency of VPCs on the Holter were also evaluated.

### Statistical Methods

#### Power Analysis

An a priori power analysis was undertaken to determine the group sizes required for the study. It was estimated on the basis of previously published data that the median time to the primary endpoint for the placebo group would be 350 days.^b^ The period over which recruitment was initially expected to occur was 1 year and the follow-up period 2 years. In the absence of any prior data from which to estimate the size of the treatment effect, the median time to the primary endpoint in the treatment group was estimated to be 800 days. To have an 80% power of detecting a difference between groups with a type I error of 5%, it was estimated that there would need to be 32 dogs in each group. With an anticipated 10% of patients lost to follow-up the sample size required was calculated to be 36 dogs in each group.

#### Interim Analysis

An interim analysis was not originally planned as part of the study protocol. However, because of a prolonged recruitment period and the resulting increased duration of the study, an interim analysis of the outcome with respect to the primary endpoint was undertaken 44 months after recruitment to the study began. The interim analysis was performed by an author (AB) removed from recruitment, data collection, and evaluation of enrolled dogs, and was undertaken without unblinding. The results of the interim analysis were not revealed to investigators. Before the interim analysis, actions that would be taken in the light of particular outcomes were defined. The 2 treatment groups were only compared with respect to the time to primary endpoint by Kaplan Meier plots, log-rank test, and Cox proportional hazard analysis. If a difference between groups had been found to be significant at the *P* < .05 level, the individual performing the analysis would have been unblinded. If the difference, significant at the *P* < .05 level, had been demonstrated in favor of the placebo group, the trial would have been stopped on the basis of safety concerns. If the difference between groups had been significant and in favor of the treatment group at the *P* < .001 level, the trial would have been stopped because of superiority of treatment.^14^ No prior criteria for futility were defined.^14^

#### Final Analysis

Data were examined by visual plots and appropriate statistical tests to determine whether or not they were normally distributed and had equal variances (Shapiro-Wilk test and Levene's test, respectively).

Baseline characteristics of the groups were compared for homogeneity. Continuous variables that were normally distributed were compared between the groups by unpaired Student's *t*-tests. Continuous variables that were not normally distributed were compared between groups by the Kruskal Wallis test. Proportions were compared by the Chi-square or Fisher's exact test.

Time to event data including time to primary endpoint and time to the secondary endpoint were compared between treatment groups by Kaplan Meier plots and log-rank tests.

The influence of treatment and baseline variables on outcome was estimated by Cox proportional hazards analysis. In these analyses, LVIDD and LVIDS were normalized for body weight (LVIDDN and LVIDSN, respectively).^15^ Baseline variables considered in the univariable analyses were as follows: age, sex, body weight, heart rate on physical examination, the presence of any arrhythmia on the 3-minute ECG, the total number of VPCs on the 3-minute ECG, the presence of 4 or more VPCs on the 3-minute ECG, the total number of VPCs on the 24-hour ECG, the presence of a complex arrhythmia on the 24-hour ECG, LVIDSN, LVIDDN, and treatment.

Variables that showed a significant association with the time to primary endpoint at the 20% level in the univariable analysis were taken forward into a multivariable model. Because there were 5 variables that related to the presence or absence of arrhythmias, only 1 arrhythmia-related variable was entered into the model. The variable chosen for the model was the presence of ≥4 VPCs on the 3-minute ECG, because this was the variable by which randomization had been stratified. Two of the variables were measures of heart size and therefore only one of these was entered into the model. Normalized systolic diameter was chosen as systolic diameter was the main determinant of inclusion to the study. Analyses were run in a backward stepwise manner and the variable with the highest *P* value was eliminated from the model until all variables in the model had a *P* value < .05. The sensitivity of the final explanatory model to the arrhythmia-related variable chosen was tested by rerunning the analysis with each other arrhythmia variable (the presence of any arrhythmia on the 3-minute ECG, the total number of VPCs on the 3-minute ECG, the total number of VPCs on the 24-hour ECG, and the presence of a complex arrhythmia on the 24-hour ECG) substituted into the final model in place of “≥4 VPCs.” The sensitivity of the final model to the heart size variable chosen was tested by substituting LVIDDN for LVIDSN.

After a model was created in which only significant variables remained, first order interactions were examined between all possible pairs of variables remaining in the model. Interaction terms were created for each pair of variables and these were then entered into the model 1 at a time to see if the interaction term had a significant effect. In the event of a significant interaction being discovered, the interaction between the 2 variables was explored further and the nature of interaction accounted for in the final model.

The influence of treatment on echocardiographic variables was examined in the following ways. The LVIDD (mm) and the LVIDS (mm) obtained at the examination before enrollment were subtracted from the value of the same variable obtained at the examination performed approximately 1–2 months after inclusion. This created the variable delta D (ΔD, mm) for the change in diastolic diameter and delta S (ΔS, mm) for the change in systolic diameter. Values for ΔD and ΔS were compared between treatment groups by an unpaired Student's *t*-test.

The influence of the magnitude of the change in heart size on outcome was explored with a second, exploratory, multivariable Cox proportional hazards model. The change in systolic diameter, LVIDSN, and treatment group were entered into this model.

The influence of treatment on the number of VPCs on the Holter was examined in the following way. The total number of VPCs over 24 hours + 1 was log-transformed to create the normally distributed variable “Log10 (VPC 24 hours + 1).” The number 1 was added to avoid the attempted log transformation of zero. For all dogs for which preinclusion and 2nd visit Holters were available, paired *t*-tests were used to compare the number of VPCs before and after the introduction of treatment in both treatment groups. The proportions of dogs in each group considered to have a complex ventricular arrhythmia were compared before and after the introduction of treatment by a Fisher's exact test.

A *P* value <.049 was considered significant for the log-rank test with respect to the primary endpoint. This adjustment was because of the conduct of an interim analysis with respect to the primary endpoint of the study for which a *P* value of .001 would have been considered evidence of efficacy. For all other statistical analyses, *P* < .05 was considered significant.

Statistical analyses were conducted by various computer software packages.^g^

### Data Management

Data management was undertaken by an independent data management company.^h^ All clinical and dispenser records were collected from all centers. Data were verified and double entered into a database by separate individuals. During data entry, the 2 sets of data were compared to verify accuracy of entry of data and any discrepancies between the 2 databases were explored and resolved. Blinding was maintained during data entry and audit. Decisions on censoring and exclusions from the study were made before unblinding. Unblinding took place only after the database was locked and an independent statistician had provided the final statistical report.^i^

In preparation of the manuscript, authors followed the recommendations given in the CONSORT statement for reporting randomized clinical trials.^16^ This was done to improve the clarity of the study aim and the transparency of the study conduct, and to improve the ability of the reader to assess the validity of the results.

## Results

Recruitment to the study began in July 2006 and finished in December 2010. Follow-up was continued for a further 10 months after recruitment had been completed. An interim analysis was undertaken in February 2010 at which time 60 dogs had been recruited to the study, of which 27 had reached the primary endpoint. The criterion for unblinding was not met.

**Fig. 1:**
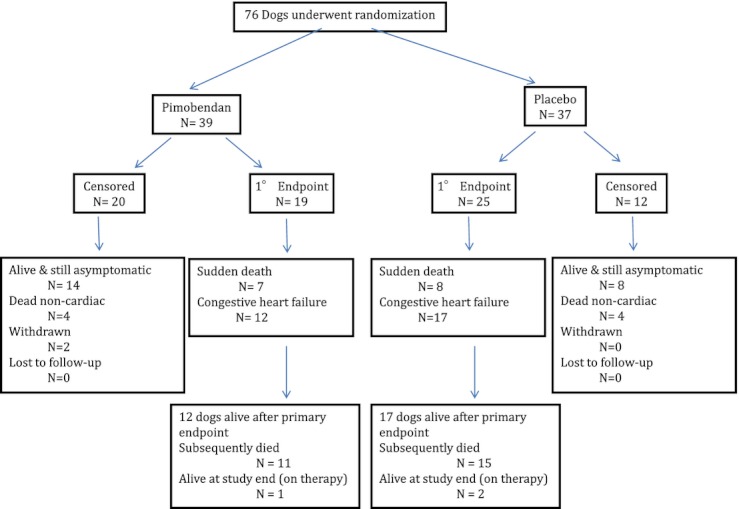
A flow chart indicating the outcome for the 76 dogs enrolled in the study.

Approximately, 1,000 dogs were screened to determine whether they met the entry criteria of the study. Seventy-six dogs were recruited to the study. The outcome after recruitment for these 76 dogs is summarized in Figure 1. The median time in the study for all recruited dogs was 427 days. Forty-four dogs reached the primary endpoint, resulting in an overall event rate for the study of 58%. The median time in study for dogs reaching the primary endpoint was 373.5 days. Ten dogs died or were withdrawn from the study for other reasons. The median time in study for these dogs was 445 days. Twenty-two dogs were still alive and had not reached the primary endpoint at the time of closure of the study. The median time in study for these dogs was 529 days. No dogs were lost to follow-up.

Thirty-nine dogs were randomized to receive pimobendan and 37 were randomized to receive placebo. In the group administered pimobendan, the median dose received was 0.453 mg/kg/day (interquartile range (IQR), 0.310–0.503 mg/kg/day)^c^^.^ Analyses for homogeneity of groups showed no significant difference between groups for any of the characteristics measured at baseline (Table 2).

**Table 2:** Selected baseline characteristics in the 2 treatment groups expressed as frequencies and (proportions%) or medians and [interquartile range]

		Treatment Group		
			
	Variable	Pimobendan (n = 39)	Placebo (n = 37)	*P* Value
Dog characteristics	Age (years)	7.08 [5.9–8.4]	7.17 [6.0–8.0]	*P* = .96
	Sex: Male/Female	18/21 (46%/54%)	17/20 (46%/54%)	*P* = .50
	Body weight (kg)	36.0 [33.0–40.0]	34.5 [32.6–42.1]	*P* = .88
Physical examination	Heart rate (beats/minute)	110 [100–130]	109 [91–120]	*P* = .15
Thyroid status	Euthyroid/hypothyroid	37/2 (95%/5%)	34/3 (92%/8%)	*P =* .61
ECG (3 minutes)	Arrhythmia on 3-minute ECG: (yes/no)	11/28 (28%/72%)	11/26 (30%/70%)	*P* = 1.00
	Total No. VPCs/3 minutes	0 [0–2]	0 [0–1.5]	*P* = .71
	≥4 VPCs/3 minutes (yes/no)	8/31 (21%/79%)	5/32 (14%/86%)	*P* = .54
24-hour Holter monitor	No. VPCs/24 hours	14.5 [3.75–372.3]	56.0 [6.5–265.5]	*P* = .55
	Arrhythmia complexity (yes/no)	3/36 (8%/92%)	6/31 (16%/84%)	*P* = .30
Echocardiography	Normalized LVIDS	1.40 [1.33–1.56]	1.43 [1.36–1.57]	*P* = .29
	Normalized LVIDD	1.77 [1.72–1.94]	1.81 [1.73–1.94]	*P* = .63

VPC, ventricular premature complex; LVIDS, left ventricular internal dimension in systole; LVIDD, left ventricular internal dimension in diastole.

In the placebo group, 25 dogs reached the primary endpoint, 4 dogs died or were withdrawn for other reasons, and 8 dogs were alive at the end of the study. In the pimobendan group, 19 dogs reached the primary endpoint, 6 dogs died or were withdrawn for other reasons, and 14 dogs were alive at the end of the study (Table 3). The proportions of dogs reaching the primary endpoint were not significantly different between groups (*P* = .1).

**Table 3:** Reasons for Censoring of 32 Dogs by Treatment

		Treatment Group		
			
		Pimobendan	Placebo	Total
Euthanasia (noncardiac)	Subtotal	4	4	8
	Neurological signs/spinal disease	2	0	2
	Abdominal hemorrhage, suspected splenic mass	0	1	1
	Gastric volvulus	0	1	1
	Gastrointestinal obstruction	0	1	1
	Mammary carcinoma	1	0	1
	Osteosarcoma	0	1	1
	Possible lymphoma	1	0	1
Noncompliance	Subtotal	2	0	2
	Owner noncompliance	1	0	1
	Removal by investigator, suspected pulmonary neoplasm	1	0	1
Alive at the end of the study	Subtotal	14	8	22
	Total	20	12	32

**Fig. 2:**
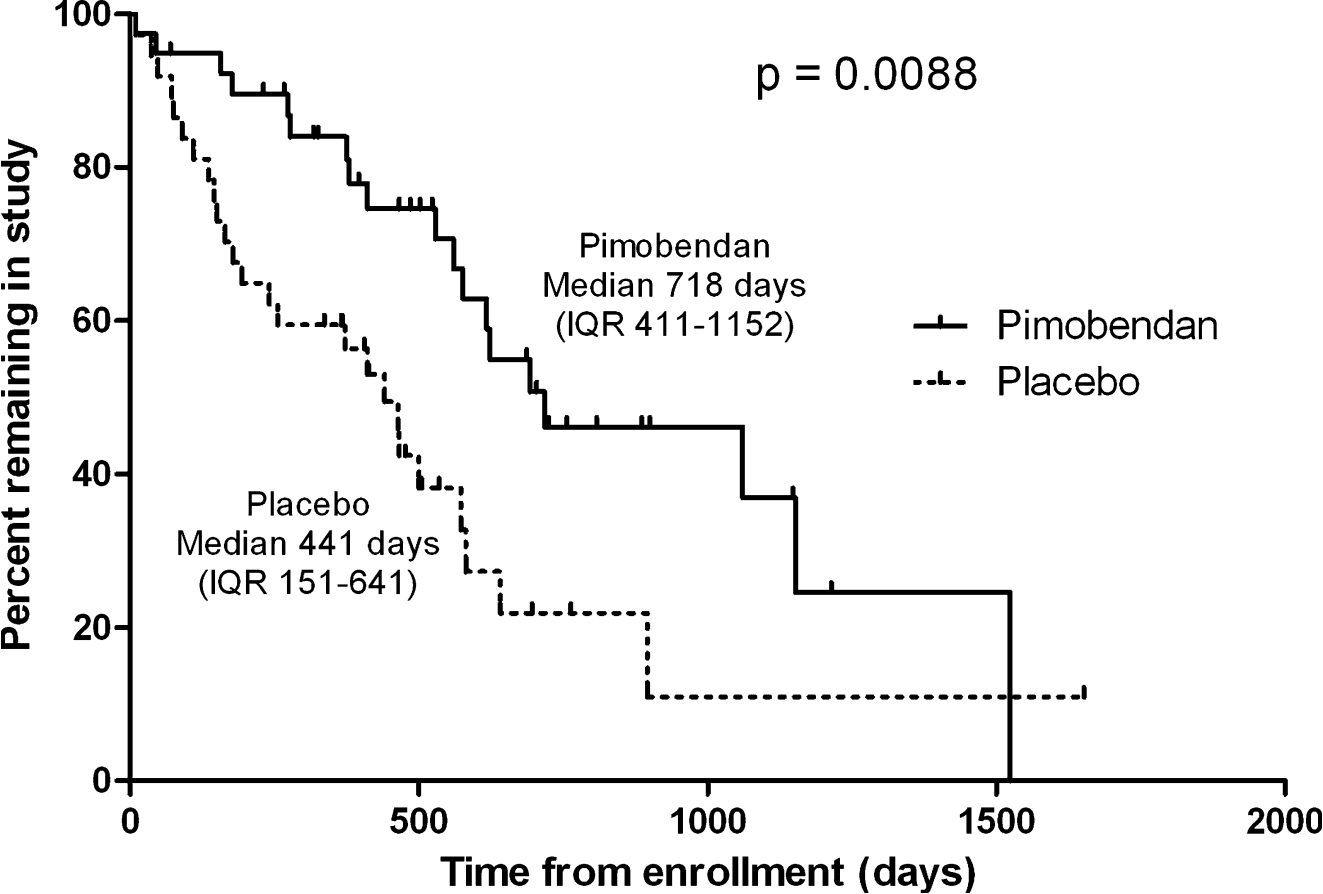
Kaplan Meier survival curves plotting the estimated percentage of dogs in each group that have not yet met the primary endpoint, against time. IQR, interquartile range

The estimated median time to the primary endpoint was significantly longer for dogs receiving pimobendan (718 days, IQR 441–1152 days) than for dogs receiving placebo (441 days, IQR 151–641 days) (log-rank *P* = .0088) (Figure 2). The median time to reach the primary endpoint for dogs in the pimobendan group was 63% (9 months) longer than for those in the placebo group. When a subanalysis was performed including only euthyroid dogs, the results were similar. The estimated median time to the primary endpoint was significantly longer for dogs receiving pimobendan (693 days, IQR 441–1,152 days) than for dogs receiving placebo (441 days, IQR 146–641 days) (log-rank *P* = .018). Too few hypothyroid dogs (n = 5) were included in the study to allow their analysis as a separate subgroup.

Of the 19 dogs reaching the primary endpoint in the pimobendan group, 12 (63%) developed CHF and 7 (37%) died suddenly. Of the 25 dogs in the placebo group that reached the primary endpoint, 17 (68%) developed CHF and 8 (32%) died suddenly. The proportion of dogs reaching the different components of the primary endpoint in the different groups was not significantly different (*P* = .76). Comparing only those dogs that reached each subendpoint, the median time to each subendpoint was not significantly different between groups (Table 4).

**Table 4:** The number of dogs in each treatment reaching the component parts of the primary endpoint and the median [IQR] time taken to do so. CHF, congestive heart failure. The comparison between groups compares the time to subendpoint only for those dogs that reached that subendpoint

	Treatment group		
		
	Pimobendan	Placebo	*P* Value for Comparison (Mann Whitney)
Number reaching primary endpoint	19	25	
Subendpoint: Onset of CHF	N = 12	N = 17	*P* = .14
Median [IQR] time in days to that endpoint.	395 [274.3–969]	256 [130.5–536.5]	
Subendpoint: Sudden cardiac death	N = 7	N = 8	*P* = .15
Median [IQR] time in days to that endpoint.	576 [45–623]	141 [54.75–320.3]	

**Fig. 3:**
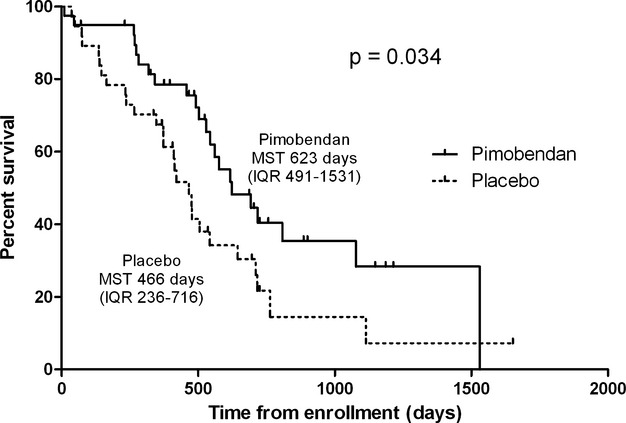
Kaplan Meier survival curves for the all-cause mortality analysis, plotting the estimated percentage of surviving dogs in each group, against time. MST, median survival time; IQR, interquartile range.

Kaplan Meier plot illustrating the estimated proportion of dogs in each group remaining alive (all-cause mortality) is illustrated in Figure 3. The estimated median survival time was significantly longer for dogs receiving pimobendan (623 days, IQR 491–1,531 days) compared with dogs receiving placebo (466 days, IQR 236–710 days) (log-rank *P* = .034).

**Fig. 4:**
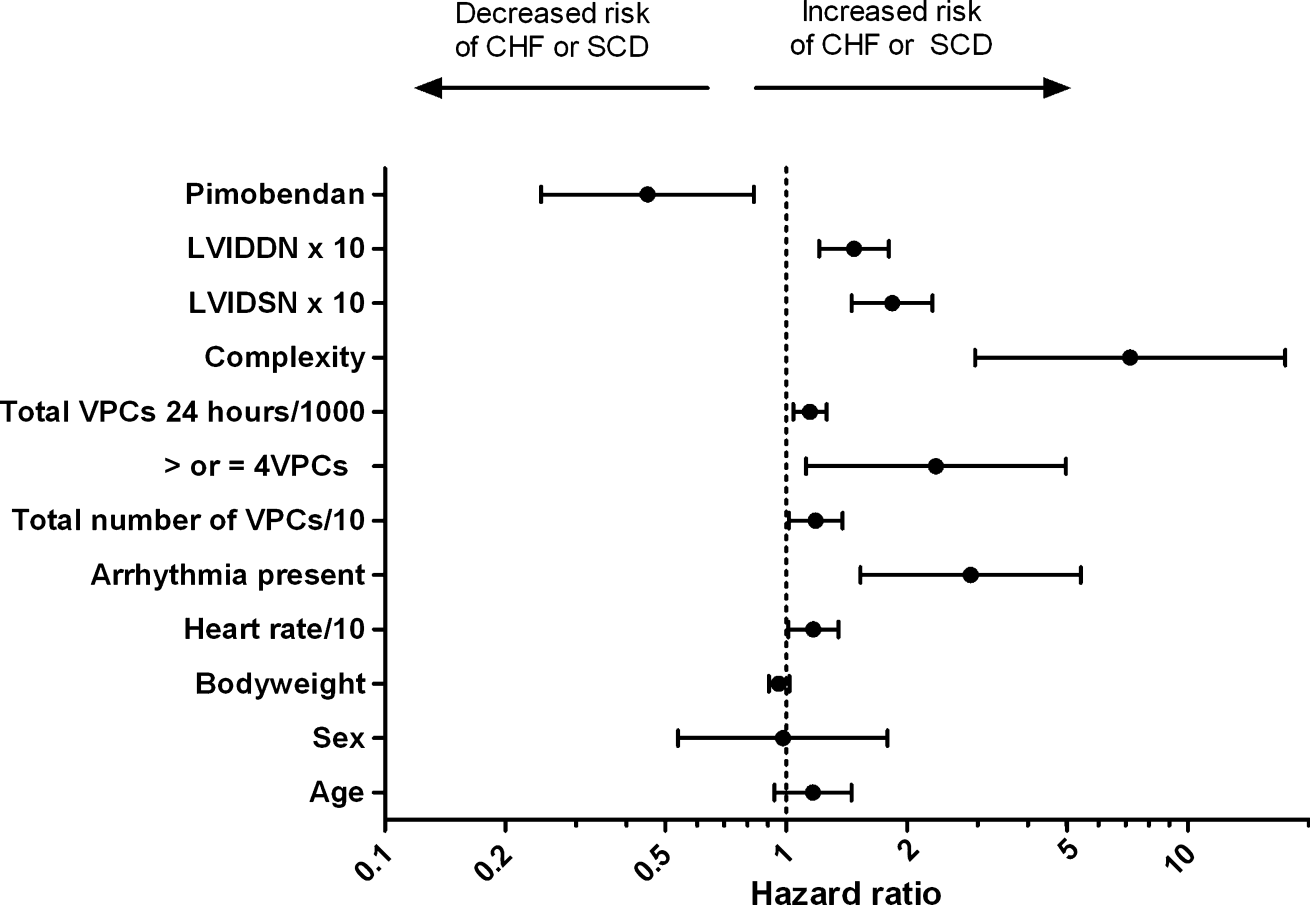
A forest plot showing the hazard ratio and 95% confidence intervals associated with variables considered in the univariable analyses with time to the primary endpoint (congestive heart failure or sudden death) as the dependent variable. Circles represent the hazard ratio and the horizontal bars extend from the lower limit to the upper limit of the 95% confidence interval of the estimate of the hazard ratio. CHF, congestive heart failure; SCD, sudden cardiac death; LVIDDN, normalized left ventricular internal diameter in diastole; LVIDSN, normalized left ventricular internal diameter in systole; VPCs, ventricular premature complexes; /10 indicates that the hazard ratio is for a 10-unit change in the variable of interest; /1000 indicates that the hazard ratio is for a 1000-unit change in the variable of interest; ×10 indicates that the hazard ratio is for a 0.1-unit change in the variable of interest.

Univariable analysis showed that there was an increase in the hazard of reaching the primary endpoint in dogs with a greater LVIDSN (hazard ratio (HR) = 1.836 (95% confidence intervals (CI) = 1.457, 2.315) for a 0.1 unit increase in LVIDSN) and a greater LVIDDN (HR = 1.482 (95% CI = 1.213, 1.810) for a 0.1 unit increase in LVIDDN) (Fig 4). There was also a significant increase in the hazard of reaching the primary endpoint in dogs with higher heart rates on physical examination (HR = 1.17 per 10 bpm increase in heart rate; 95% CI = 1.01, 1.35) and evidence of arrhythmias (the presence of any arrhythmia [if an arrhythmia present; HR = 2.88; 95% CI = 1.53, 5.41] on the resting ECG; greater number of VPCs on the resting ECG [if ≥4 VPCs; HR = 2.36; 95% CI = 1.12, 4.97]; and greater numbers of VPCs on the Holter recording [HR = 1.146 for each 1000 VPCs on the Holter ECG; 95% CI = 1.041, 1.261] were all associated with a worse outcome) (Fig 4).

The explanatory multivariable model created after the backward stepwise process, but before consideration of interaction terms, contained 4 variables. These were treatment group, LVIDSN, baseline heart rate, and whether or not a dog had ≥4VPCs. The effect of treatment remained significant when all other arrhythmia variables were substituted for ≥4 VPCs in the final model and when normalized diastolic diameter was used in the model instead of normalized systolic diameter.

Six interaction terms were created examining the interactions between each of the possible pairs of variables in the model. There was no significant interaction between treatment and any of the other variables. The only significant interaction was between heart rate and whether or not a dog had ≥4VPCs. Dogs were therefore categorized into 4 groups according to whether or not they had a heart rate above or below the median (110 bpm) at baseline, and whether or not they had ≥4 VPCs. Group 1 consisted of 32 dogs with a heart rate <110 bpm and <4 VPCs; this group was used as the referent category in the multivariable analysis. Group 2 consisted of 4 dogs with a heart rate <110 bpm and ≥4 VPCs. Group 3 consisted of 31 dogs with a heart rate ≥110 bpm and <4 VPCs. Group 4 consisted of 9 dogs with a heart rate ≥110 bpm and ≥4 VPCs.

The significant effect of treatment in prolonging the time to the primary endpoint persisted after adjustment for the influence of other variables (Table 5). The hazard ratio from the multivariable analysis for the effect of treatment implies that dogs in the placebo group were 3.3 times more likely than those in the pimobendan group to reach the primary endpoint first. The hazard of reaching the primary endpoint approximately doubled for every 0.1 unit increase in normalized systolic diameter. Dogs with ≥4 VPCs and an above median heart rate were approximately 7 times more likely to reach the primary endpoint first.

**Table 5:** Explanatory multivariable model. HR, hazard ratio; CI, confidence intervals

			95.0% CI for HR	
			
Variable	Sig. *P* =	Hazard ratio (HR)	Lower	Upper
Treatment (pimobendan compared with placebo)	<.001	0.299	0.153	0.585
Normalized left ventricular systolic diameter (HR for 0.1 unit increase)	<.001	2.024	1.568	2.614
Overall effect of VPCs and heart rate	.005			
< 4 VPC and heart rate <110 (reference)		1		
≥4 VPC and heart rate <110	.60	1.406	0.398	4.961
< 4 VPC and heart rate ≥110	.23	1.543	0.761	3.129
≥4 VPC and heart rate ≥110 compared to	<.001	7.044	2.436	20.370

**Fig. 5:**
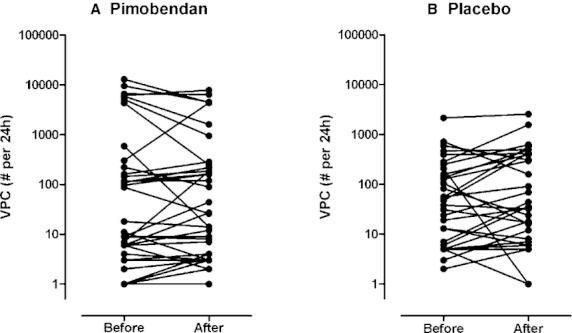
The number of VPCs on the 24-hour ECG before and approximately 1 month after the initiation of treatment in the group receiving pimobendan (A) and the group receiving placebo (B). Differences were not significant in either group. VPC, ventricular premature complex. Four datapoints do not appear on the logarithmic scale when the VPC number was zero. These are 2 dogs in the pimobendan group pretreatment, 1 pimobendan dog post treatment, and 1 placebo dog pretreatment.

Paired Holter data were available for 70 dogs: 37 in the pimobendan group, and 33 in the placebo group. All repeat Holters were recorded between 20 and 56 days after initiation of treatment. The median time to the 2nd Holter recording was 30 days (IQR 27–38.5 days) for the pimobendan group and 33 days for the placebo group (IQR 29–38 days). The time to 2nd recording was not significantly different for the groups (*P* = .33). Reasons for missing data were as follows: 1 pimobendan dog had sudden cardiac death; the other missing pimobendan dog had absent data because of technical difficulties at baseline; 1 placebo dog had sudden cardiac death; 1 placebo dog had developed CHF; 1 placebo dog did not have a 2nd Holter ECG until over 1 year after initiating treatment; and 1 placebo dog had absent data because of technical difficulties. In neither group was there a significant change in VPCs after treatment [comparison of log_10_ (total VPC + 1) pimobendan group *P* = .93, placebo group *P* = .33] (Fig 5A and 5B). Three of the pimobendan dogs were considered to have complex ventricular arrhythmias before treatment and 5 to have complex ventricular arrhythmias afterward (the original 3 dogs and 2 additional dogs). Six of the placebo dogs were considered to have complex ventricular arrhythmias before treatment and 6 to have complex ventricular arrhythmias afterward. (The 6 dogs with complex arrhythmias at the 2nd examination consisted of 4 of the original 6 dogs and 2 dogs not considered to have complex arrhythmias on the 1st examination. Of the 2 dogs considered to have complex arrhythmias at the original examination, but not at the 2nd examination, 1 dog had died and the other was considered not to have a complex arrhythmia on the 2nd Holter examination.) None of the dogs received any antiarrhythmic treatment between Holter examinations.

**Fig. 6:**
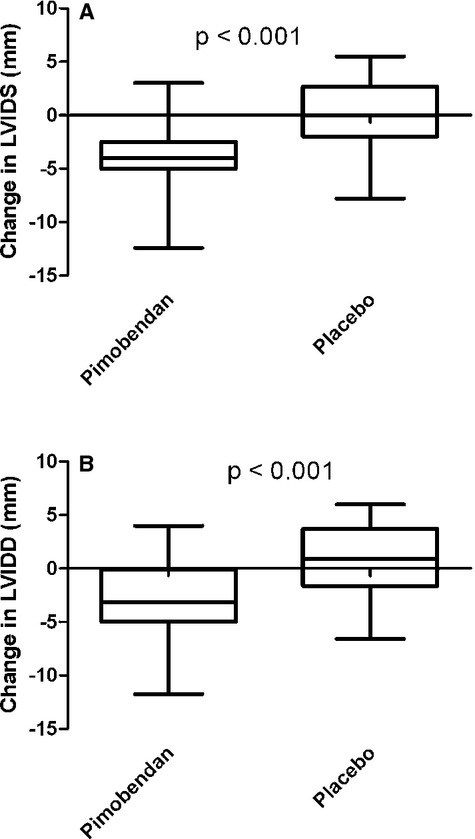
The change in left ventricular diameter occurring within the first 2 months of the study for dogs in which paired observations were available (73 dogs, 38 receiving pimobendan, and 35 receiving placebo). The change in systolic diameter is illustrated in Figure 6A and the change in diastolic diameter in Figure 6B. LVIDS, left ventricular internal diameter in systole; LVIDD, left ventricular internal diameter in diastole.

Paired echocardiographic data were available for 73 dogs: 38 in the pimobendan group, and 35 in the placebo group. Reasons for failure to obtain a 2nd echocardiographic examination were, in all cases, that the dogs had already reached the primary endpoint. All repeat echocardiographic examinations were conducted between 20 and 56 days after the initiation of treatment. The median time to 2nd examination for the group receiving pimobendan was 30 days (IQR 27–37 days) and for the group receiving placebo was 34 days (IQR 30–38.5 days); the difference in time to the 2nd recording for the 2 groups was not significant (*P* = .18). The changes in LVIDS and LVIDD from the 1st echocardiographic examination to the 2nd examination are illustrated for both groups in Figure 6. The median change in LVIDS in the pimobendan group was −4 mm [−5.0 to −2.48 mm]. The median change in LVIDS in the placebo group was 0 mm [−2.0 to 2.7 mm] (*P* < .001 for the comparison between groups). The median change in LVIDD in the pimobendan group was −3.1 mm [−5.0 to −0.07 mm]. The median change in LVIDD in the placebo group was 0.9 mm [−1.6 to 3.7 mm] (*P* < .001 for the comparison between groups).

Sotalol treatment was administered to 8 dogs in total, one of which also received mexiletine. Sotalol treatment was introduced to 2 dogs in the placebo group 130 days and 146 days after randomization. Sotalol treatment was introduced to 6 dogs in the pimobendan group, a median of 242.5 days (range 53–935 days) after randomization.

Results of the exploratory multivariable analysis examining the influence of LVIDSN, change in LVIDS, and treatment on the time to the primary endpoint are illustrated in Table 6.

**Table 6:** Exploratory multivariable model. LVIDSN, normalized left ventricular internal diameter in systole at baseline; HR, hazard ratio; CI, confidence intervals; ΔS, change in left ventricular diameter

			95.0% CI for HR	
			
Variable	Sig. *P* =	Hazard Ratio	Lower	Upper
Treatment (Pimobendan compared with placebo)	.13	0.565	0.272	1.172
Baseline LVIDSN (HR for a 0.1-unit change)	<.001	1.841	1.404	2.416
ΔS (mm)	.005	1.164	1.047	1.293

None of the suspected adverse reactions or potential complications resulted in the withdrawal of a dog from the study (Table 7).

**Table 7:** Suspected adverse drug reactions (not leading to withdrawal in 76 dogs)

	Treatment Group	
	
Suspected Adverse Drug Reactions	Pimobendan	Placebo
Gastrointestinal disorders (eg, emesis, diarrhea, inappetence)	6	3
Abnormal behavior (eg, lethargy, confusion, restlessness)	1	2
Head bobbing (transient) after administration of mexiletine, condition resolved	1	
Increased respiratory rate and effort with exercise		1
Cough (unexplained cause)		1
Mast cell tumor, left thorax		1
Pulmonary mass and rectal mass		1
Ventricular arrhythmia on Holter monitor recording	1	
Irregular and rapid heartbeats reported by owner		1
Syncope		4
		
Total	9	14

## Discussion

This study demonstrates a survival benefit of the chronic administration of pimobendan initiated in the preclinical phase of a naturally occurring cardiac disease in Dobermans. Pimobendan administered to Dobermans with preclinical DCM prolonged, by 9 months, the median time to the onset of CHF or sudden death and prolonged the time to death attributable to all causes.

Screening dogs and cats for preclinical cardiac disease has become commonplace; however, despite this practice, no previous prospective study has been able to fulfill the criterion of demonstrating the effectiveness of a treatment administered in the early stage of the disease.^17^ In this study, a large population of apparently healthy Dobermans was screened to identify dogs with echocardiographic evidence of DCM.

There is limited literature addressing the merits of treatment of patients with preclinical left ventricular systolic dysfunction, in both humans and domestic animals. The 2009 update to the guidelines for the management of adults with heart failure recommends the use of ACE inhibitors and beta receptor blockers to retard the progression of left ventricular systolic dysfunction.^6^ In the veterinary literature, even less attention has been focused on attempting to slow the progression of cardiac remodeling by initiating treatments during the preclinical stage of heart disease. Two prospective studies assessed the merit of treating MMVD before the onset of signs.^18^,^19^ Both studies demonstrated no difference between the treatment group (enalapril) and the placebo group with respect to significantly delaying the onset of clinical signs of heart disease—the primary endpoint in both studies. More recently, and after commencement of the study described in this report, a retrospective study suggested a significant delay in the time to onset of CHF or sudden death for Dobermans with preclinical DCM that were treated with the ACE inhibitor benazepril.^7^

Previous studies demonstrating the harmful effects of some inotropic agents in human heart failure patients^20^,^21^ resulted in concern among veterinary cardiologists that there may be similar detrimental effects associated with the chronic administration of inotropic agents in dogs. There is no evidence in the veterinary literature that pimobendan administration poses a risk to dogs with DCM. This study demonstrated no increased risk of proarrhythmia or sudden death associated with pimobendan use in a cohort of Dobermans with preclinical DCM. Furthermore, pimobendan had a favorable effect on all-cause mortality. In addition, neither treatment group showed any significant alteration in the frequency of VPCs assessed over a 24-hour period comparing recordings obtained before and approximately 1 month after initiation of treatment. Taken together these data provide evidence to suggest that pimobendan treatment in a cohort of Dobermans with preclinical DCM was not associated with any worsened arrhythmia.

Although the primary purpose of any clinical trial is to establish the effectiveness of an intervention, a secondary benefit of such studies is the ability to study the effects of other factors recorded at baseline on the outcome of interest in a well-characterized population. The ability of a study to do this is limited by the size of the population of dogs being studied and the number of dogs in the study that experience the outcome of interest. In this study, there were 76 dogs of which 44 reached the primary endpoint. By a commonly applied guideline,^22^, this should mean that we are able to determine the effect of approximately 3 other variables, in addition to treatment, on the outcome of interest. The study therefore also allowed us to demonstrate that a reduction in systolic function (indicated by an increase in LVIDSN), a higher heart rate on physical examination, and the presence of at least 4 VPCs on a 3-minute ECG strip independently predicted the time to the primary endpoint. There was an interaction between heart rate and number of VPCs whereby the dogs at highest risk were those with higher than median heart rate and 4 or more VPCs. These dogs were approximately 7 times more likely to reach the primary endpoint first compared with those with lower than median heart rate and fewer than 4 VPCs. Important to the primary objective of the study, the beneficial effect of pimobendan persisted after adjusting for the effects of these other variables.

In univariable analyses, this study demonstrated that numerous indicators of the presence of arrhythmias, whether at least 4 VPCs on a 3-minute ECG or the frequency of VPCs per 24 hours (Holter), were associated with an adverse outcome; either onset of CHF or sudden death. Others have previously reported the adverse association of VPCs and outcome in dogs and humans with DCM.^5^,^23^ In addition, this study showed that if a dog had any arrhythmia, supraventricular, or ventricular, they had a worse outcome when compared with dogs with no detectable arrhythmia. Lastly, this study showed the clinical relevance of a simple 3-minute ECG trace to predict outcome; the presence of 4 or more VPCs on the 3-minute ECG trace increased the risk of reaching the primary endpoint approximately 2.5-fold.

In this study, LVIDS and LVIDSN were used as surrogates to assess left ventricular systolic function and reductions in systolic function were associated with an adverse outcome. This finding is in agreement with others^5^ and has been well demonstrated in humans with systolic dysfunction.^24^

Dobermans have limited genetic variability^25^ that permits the examination of a relatively homogenous population of dogs with preclinical DCM. Such homogeneity of disease expression and progression allows the use of a relatively small sample size to demonstrate a significant treatment effect by eliminating confounding factors that might be found in a more heterogeneous population. However, it poses questions regarding the generalizability of this study's findings to preclinical DCM in other large breed dogs. Interim results of an ongoing study have already suggested encouraging evidence of a benefit of pimobendan treatment in Irish Wolfhounds with occult DCM.^j^ Although it has previously been suggested that DCM in Dobermans might be different to DCM in other breeds, one recent large retrospective study indicated that after adjusting for the degree of systolic dilatation, presence of left-sided congestive heart failure, and ventricular arrhythmia frequency (among other variables), Dobermans did not have a significantly different prognosis compared with other breeds with DCM.^5^ The progression of DCM in dogs and the similarities in phenotype, pathology, and pathophysiology associated with DCM across breeds suggest that there is a potential for similar benefit associated with the administration of pimobendan in the preclinical phase of DCM in other breeds, although further prospective studies would be required to demonstrate this.

The main criterion used to identify Dobermans with preclinical DCM was an elevated LVIDS. The values used were derived from studying a normal population of Dobermans and although these were unpublished at the time of writing the protocol, similar criteria have subsequently been described in another study.^7^ The validity of this criterion was confirmed by the subsequent progression of the disease in a high proportion of the included dogs to the prespecified primary endpoint. LVIDS measurements were obtained from M-mode echocardiographic views and more recently published data have suggested that other echocardiographic techniques than M-mode may be superior in the early identification of Dobermans with DCM.^26^

Other studies have demonstrated that Dobermans can present with only ventricular premature complexes as an indication of the presence of preclinical DCM.^1^ As all dogs in our study demonstrated evidence of systolic dilatation at the time of enrollment, we cannot extrapolate the results of our study to dogs that present with VPCs in the absence of systolic dilatation.

There were several exclusion criteria for enrollment in this study. Dogs with atrial fibrillation or malignant ventricular arrhythmias were excluded. In addition, dogs with significant comorbidities such as renal failure, hepatic failure, and some endocrine disorders were excluded. A small number (n = 5) of dogs with stable hypothyroidism were included. Censoring hypothyroid dogs from analyses of the reported endpoints did not significantly change the results of the analysis or conclusions. These entry criteria permitted the inclusion of the majority of Dobermans with preclinical DCM as evidenced by our screening data. Nevertheless, the restricted selection in the enrollment necessitates that caution is exercised when extending our conclusions to dogs that meet any of our exclusion criteria.

A number of study design factors, in addition to those discussed above, increased the potential of this study to determine the effect of treatment. The dogs were randomized to ensure equal representation by sex and by frequency of VPCs. Although sex was not subsequently shown to have a significant influence on outcome, the presence or frequency of VPCs was significant even after adjusting for the influence of treatment and degree of systolic dilatation in the multivariable analysis. Many human and veterinary clinical trials suffer from low event rates (therefore high censoring rates). The duration of this study (5.3 years and median time in study of 427 days) increased the event rate and augmented the power of this study to demonstrate a treatment effect.

Analysis of the follow-up echocardiographic data showed that the administration of pimobendan led to a significant reduction in LVIDS that was associated with a positive outcome or reduction in the hazard for development of the primary endpoint. The reduction in LVIDS and LVIDD could represent a combination of augmentation of systolic function, reduction in afterload, reverse remodeling, or a combination of all three of these factors. Whatever the cause of the reduced LVIDS, the exploratory multivariable analysis showed that the change in LVIDS was highly predictive of subsequent outcome even when the original LVIDSN was taken into account. The observation that the baseline LVIDS and subsequent change in systolic diameter were more predictive of outcome than the treatment group in this analysis suggests that this alteration of ventricular size may be one of the primary ways in which pimobendan mediates its favorable effects in this population.

One of the primary requirements for the administration of any medication, particularly in the preclinical setting, is that it is safe. This study collected data on suspected adverse drug reactions (SADRs). There was no clinically relevant difference in the frequency or severity of SADRs between the 2 treatment groups, thereby providing additional evidence of the safety of pimobendan in this cohort of dogs.

This study did not follow the outcome for the dogs that were screened, but not enrolled in the study. Although this was not a primary objective of the trial, such data would have permitted a better analysis of the impact of screening on the population as a whole.

## Conclusions

The administration of pimobendan significantly prolonged, by 9 months, the median time to the onset of CHF or sudden death in Dobermans with preclinical DCM and also resulted in prolongation of the time to death attributable to all causes. This beneficial effect persisted after adjusting for other baseline variables. The administration of pimobendan did not increase the presence of, or frequency of, VPCs or sudden death in the treatment cohort.

Greater dilatation of the left ventricle in systole, heart rate on physical examination, and having at least 4 VPCs on a 3-minute ECG strip were independent predictors of adverse outcome after adjusting for the effect of treatment.

Dogs treated with pimobendan demonstrated a significant reduction in heart size after 30 days. The degree to which heart size reduced was predictive of the time to onset of congestive heart failure or sudden death with greater reductions in size associated with better outcome.

We believe that our results represent grounds to be optimistic that pimobendan could also be effective in dogs of other breeds with similar manifestations of preclinical DCM, although further studies would be required to objectively assess the merits of pimobendan treatment in other breeds with preclinical DCM, or in Dobermans meeting some of our exclusion criteria.

## Footnotes

a O'Grady MR, Horne R. Preclinical dilated cardiomyopathy: An echocardiographic and electrocardiographic study of 193 asymptomatic Doberman Pinschers. J Vet Intern Med 1992;6:112 (abstract)

b O'Grady M R, Horne R. Echocardiographic findings in 51 normal Doberman Pinschers. J Vet Intern Med 1995;9:202 (abstract)

c Vetmedin, Boehringer Ingelheim

d Trinity Biotech USA Inc, Jamestown, NY

e According to the manufacturer's specified product characteristics in the UK

f Spacelabs Lifecard CF monitors and Spacelabs Pathfinder Holter Analysis Software, Hertford UK

g PASW version 18.0 for Windows, SPSS Inc, Chicago, IL; Graphpad Prism, La Jolla, CA

h Ondax Scientific, Hondarribia, Spain

i Llorenc Badiella, Servei d'Estadistica of Universitat Autonoma de Barcelona

j Vollmar AC, Mohren NA, Fox PR. Pimobendan versus Benazepril versus Metildigoxin in Irish Wolfhounds with preclimical DCM or AF. ACVIM Proceedings, 2012, New Orleans

## Abbreviations

2D: two-dimensional
ACE: angiotensin converting enzyme
CHF: congestive heart failure
CI: confidence interval
DCM: dilated cardiomyopathy
Doberman: Doberman Pinscher
ECG: electrocardiogram
Holter: 24-hour ambulatory ECG
HR: hazard ratio
IQR: interquartile range
LVIDD: left ventricular internal diameter in diastole
LVIDDN: left ventricular internal diameter in diastole normalized for bodyweight
LVIDS: left ventricular internal diameter in systole
LVIDSN: left ventricular internal diameter in systole normalized for bodyweight
MMVD: myxomatous mitral valve disease
PROTECT: pimobendan randomized occult DCM trial to evaluate clinical symptoms and time to heart failure
SADRs: suspected adverse drug reactions
T4: thyroxine
VPC(s): ventricular premature complex(es)
ΔD: change in diastolic diameter
ΔS: change in systolic diameter
